# The evolution of our profession and association from 1998–2023: reflections from four Medical Library Association leaders

**DOI:** 10.5195/jmla.2024.1948

**Published:** 2024-07-01

**Authors:** Kristine M. Alpi, Julie M. Esparza, Brenda F. Green, Shannon D. Jones

**Affiliations:** 1 krisalpi@gmail.com, Associate Dean of Libraries & Information Sciences, Icahn School of Medicine at Mount Sinai, New York, NY; 2 julia.esparza@lsuhs.edu, Professor/Associate Director, Comegys Endowed Professor of Medical Library Science, Louisiana State University Health at Shreveport, Shreveport, LA; 3 greenbrenda67@gmail.com, Associate Professor, Retired, University of Tennessee Health Science Center, Memphis, TN; 4 joneshan@musc.edu, Director of Libraries, Medical University of South Carolina, Director, NNLM Region 2, Charleston, SC

## Abstract

On the occasion of the Medical Library Association's 125th Anniversary, four librarian leaders with a combined 105 years of engagement in MLA collaborated to reflect on the changes in our profession and our association. We draw on an examination of the last 25 years of the MLA Janet Doe Lectures, our own personal histories, and scholarship we produced for MLA publications and presentations. We offer this compilation as an invitation for readers to reflect on their experiences of changes within the profession, inspiration to engage in the issues around our place in society, and a source for additional exploration into researching and learning from our collective history.

## INTRODUCTION

The collaborative work that follows resulted from an invitation to reflect on our personal experiences during the last 25 years of the Medical Library Association (MLA). This period encompasses almost the entirety of our professional connections with MLA, ranging from 22 to 32 years each. We structured it as individual essays, but we each read and commented on each other's experiences, recognizing that we had some shared experiences during overlapping association activities and unique perspectives. Brenda Faye Green begins with an examination of the last 25 years of the MLA Janet Doe Lectures. Then, three past MLA presidents–Julie Esparza, Kristine Alpi, and Shannon Jones–offer essays on changes in our profession and our association through the lens of our personal histories reinforced by references to scholarship we produced for MLA publications and presentations. We aim to share our view of changes within the profession, inspire you to engage in the issues around our profession and association's place in society, and continue learning from our collective history.

**Brenda F. Green, FMLA, Associate Professor, Retired, University of Tennessee Health Science Center**

## JANET DOE LECTURES

My professional development as an academic health sciences librarian has been enriched through attendance at the MLA's annual meeting. These meetings are characterized by a plethora of programming, continuing education opportunities, social engagements, and various supplementary activities. Notably, the scheduling of concurrent sessions during the meeting is customary, ensuring a diverse array of topics and discussions for attendees.

However, the annual Janet Doe Lecture (Doe Lecture) is one of three lectures that do not have competing programming or activities thus signaling the significance of the lecture [1]. The Doe Lecture envisioned to address historical and/or philosophical topics, began in 1966 [2]. Early in my career I began attending the lectures.

This article aims to retrospectively examine the Doe Lectures from 1998–2023. During this time, lecturers shared interesting hobbies, commented on issues of the day, celebrated thought leaders and risk-takers, and explored historically significant topics. Diverse lecture topics were delivered.

### Diverse Topics

Throughout this period, biographical and historical lectures have covered a broad spectrum of topics. Many speakers elected to disclose personal information that was previously undisclosed or limited to their inner circle.

Rick Forsman mentioned “scuba trips” in his 2004 lecture and he gave an apt description of the lecturer's mindset when he wrote the following:

“ … the talk by its nature derives from the personal values, passions, and unique experience of the lecturer. To a significant degree it is a self disclosure, an intimate exposure of how one thinks, what one believes is important ….” [3].

Notably, Julie Sollenberger, a practitioner of mindfulness, concluded her lecture with a meditative exercise, underscoring the multifaceted nature of lectures during this time. Her lecture, along with almost all Doe Lectures, has a citation in PubMed and/or PubMed Central databases. Additionally, her citation includes a supplemental material link to her slides, and a video of her interactive lecture [4].

In 2008, Elaine Martin gave a lecture on issues of importance to her including social justice:

But I must confess my choice of topic comes from contemporary concerns about what is going on in our society today and is influenced by movements such as #BlackLivesMatter, #Metoo, #Enough, #Resistance, #NeverAgain, and #MarchforOurLives, and the proliferation of accepted terms such as “Fake News” or “alternative facts” [5].

In 2023, Michelle Kraft's historically focused lecture pointed to the actionable strategies and rationales for medical librarians to engage in efforts to combat fake news from the position of its “impact on medical and health information” [6].

In the 2011 Doe Lecture, Scott Plutchak, former editor of *Bulletin of the Medical Library Association*, provided rational for encouraging journal authors to merely state “librarians” did thus and so rather than stating our “library” completed actions done by librarians [7]. Several lecturers implored members to engage in research. One such lecture, given by Sherrilyn S. Fuller in 1999, provides a brief history of the Research Section's 1982 beginning with “Gwendolyn Cruzat, chair, Anna Cleveland, chair-elect and Dottie Eakin, secretary” [8].

### Thought Leaders and Risk-takers

Numerous speakers commended librarians demonstrating a willingness to take risks. In 2006, Julie McGowen mentioned several risk-takers in her lecture. McGowen provided ample proof to support her contention that the following librarians were bonafide risk-takers: Nancy Lorenzi, AHIP, FMLA; Nina W. Matheson, AHIP, FMLA; Naomi Broering, AHIP, FMLA; Rachael (Anderson) Goldwyn, AHIP, FMLA; Susan Crawford, AHIP, FMLA; Jacqueline Donaldson Doyle, AHIP, MLA; Lois Ann Colainni, AHIP, FMLA; Betsy Humphreys, AHIP; and Anne Kabler Robichaux [9].

Many of the aforementioned librarians, several of whom were featured as Doe lecturers, were recurrently cited across multiple Doe Lectures. McGowan's meticulously researched presentation offered biographical insights and documented pivotal advancements within our academy of practice. Their contributions to these lectures often held significant historical relevance [9].

### Historically Significant Topics

Historical lecture topics included mediated services [10], textual analysis [11], evidence-based librarianship [12], and oral histories [13]. Michael Kronenfeld's 2022 historical lecture focuses on, “the transition of the health related print Knowledge-Based Information base to the emerging digital health-related ecosystem” [14]. Our academy of practice and MLA's history are documented in many lectures.

### Relevance

Doe Lectures serve as a valuable resource for biographical and historical inquiry. I encourage you to read the lectures in their entirety. Due to space limitations, only small portions of the lectures are discussed. This article highlights the Doe Lecture as a pivotal indicator of evolving trends within the profession, a platform for identifying mentors, and a reflection of MLA's commitment to addressing contemporary societal issues. Lecturers adeptly forecasted shifts within the profession and proposed actionable solutions. Moreover, they courageously challenged librarians and the association to confront the complexities of our historical narrative.

**Julia M. Esparza, AHIP, FMLA, 2019–2020 MLA President**

## CHANGES IN OUR PROFESSION

### Changes in Technology and Resources

Technology significantly changed the interaction between health information professionals and our constituents [15–18]. While reference questions still range from basic to extremely challenging, they now come via email and chat. We have adapted and become experts in new technologies. The transition from paper to electronic resources led to innumerable changes in workflow. Personally, 25 years ago, while working as a serials librarian, I went from checking hundreds of paper journals to purchasing my first of many electronic journals, which reduced my workload and provided faster and more convenient access to library users.

Also, from Toxline to PubChem, we have witnessed the startup and decline or absorption of many health and scientific information resources. Point-of-care resources are now entrenched in our world. While some health professionals see these tools as the answer to everything, others still look to the primary literature as evidence to guide their practice—and for that, they call on us [19–20]. While there have been many times in the last 25 years that we responded rapidly to our users’ needs, never was this more vital than during the COVID pandemic when information was changing rapidly. During this period of upheaval, librarians responded by assisting in research and clinical environments with great dexterity [21–22].

### Changes in Our Environments

From 1998 to 2021, there were over 1,887 hospital mergers [23]. Mergers and consolidation into bigger systems remain a concern when they result in the elimination of health information professional positions [24]. Some downsized professionals left the profession for other opportunities. From 2002 through 2007, I was a hospital librarian. Following the complete elimination of my staff, disenchantment with hospital librarianship prompted me to move to academia. Yet, many hospital librarians thrive. Those who have flourished in hospitals and health systems have created a strong base of support by contributing to quality improvement, publishing, and systematic reviews, rounding with healthcare teams, and fulfilling other needs such as managing patient education or continuing medical education [25–28].

Hospital information professionals are not alone in responding to adversity with reinvention. Some academic health science libraries, which were once showpieces in many medical schools, have lost space—and much, if not all, of their print collections [29–37]. While going through this process, health information professionals often advocated for the best use of the space to create new learning environments. By adapting, we persevered through the changes and came out stronger. At my institution, we were hesitant to eliminate one floor of our collection to create a study area, but careful planning and design led to us having a closer connection to students.

### Changes in our Roles

In 2013, new roles for information professionals were identified—embedded librarians, informationists, systematic reviews, emerging technologies, continuing medical education, grants development and data management [38–40]. Data management ties us directly back to the research world [41–42]. Searching for datasets or to create data repositories is now a feature of some health information professionals' jobs. We help researchers organize and label their data and assist them in drafting their data management plans. This new area is helping us ensure researchers are on track to fulfill governmental and institutional regulations. However, clinical and consumer health informationists are still needed. Additionally, medical students still need to learn evidence-based medicine and researchers still need expert searching. Add this to the liaison positions, clinical librarian roles, work in the molecular and biological sciences, roles with nursing and other allied health professions, and we are a vibrant group of professionals.

Our roles have changed in a variety of ways. In the area of collection management, I have seen over the years some job titles transition from “acquisitions” and “cataloger” to “electronic services,” “electronic resource librarian,” or “digital asset manager.” Other technology roles include expanded resource management and managing 3-D printing, augmented reality, and other new technologies.

With the advent of systematic reviews, our specialized skills as health information professionals are in demand. This creates an important new role for our profession. Advocacy for health information professionals to be involved in creating high quality systematic reviews has grown since 2005 [43–45].

Leadership in the profession has also changed. While leaders still have their normal leadership duties, there is greater emphasis on ensuring equity and inclusion while addressing employee wellness and mental health issues. Library leaders must be politically savvy marketing managers, communicators, and visionaries.

Together we, as MLA, have done an amazing job helping each other deal with 25 years of profound change. Continuing education programming offered at MLA annual conference since 1998 shows a responsiveness that is essential for our profession covering topics from learning about diversity, equity and inclusion, how to complete systematic review searching, handling electronic resources, understanding coding languages, applying metadata, general new technology, and growing as a professional. In their 2023 article, Laynor, Tagge, Magro, De Armond, Rau and Vardell mentioned that MLA mentors, courses, or specializations continue to be important to developing new information health specialists [46].

As our profession changes, we answer the call through continuing education, seeking mentorship, and developing networks to hone our skills. We have faced many challenges, but we are adept at meeting them.

*Ms. Esparza greatly appreciates David C. Duggar and Elliott Freeman from Louisiana State University Health Sciences Center at Shreveport for their assistance in this piece.*

**Kristine M. Alpi, AHIP, FMLA, 2021–2022 MLA President**

## GROWING WITH MLA'S SUBJECT SPECIALIZATION AND PROFESSIONAL DEVELOPMENT NETWORKS

### Developing Expertise in Health Information Practice

During these 25 years, collections and technology have evolved, while subject specialization remains a question. Health science librarians are not defined by physical spaces and print collections, but by the learners and practitioners we connect with information. Consumer health information has evolved from curating local, volunteer-managed print and web resources to federally-funded, national services connected to corporate systems. One thing that has not changed is questioning how much subject expertise and knowledge of resources is needed to succeed within health librarianship.

I leaned into learning from MLA communities of practice. The first MLA continuing education (CE) course I attended introduced the major texts for clinical disciplines. Core lists such as the Brandon/Hill lists [47] and MLA-published BibKits were the basis of collection development. While no longer published, archived versions remain useful to identify classic texts. I used one version in 2021 to examine resources for respiratory therapists. Additionally, MLA produced print consumer health information Medspeak brochures.

### Joining MLA National and Regional Communities

MLA Chapters and Sections introduced me to exciting collaborations related to collections and publications. In 1998, I joined health and public librarians in the New York-New Jersey Chapter, collaborating on the bilingual health website New York Online Access to Health (NOAH) which won MLA's 2006 Thomson Scientific/Frank Bradway Rogers Information Advancement Award. NOAH was retired as Spanish language content became available on MEDLINEplus. In 1999, I took on an additional part-time position with nurse-turned-librarian, Susan Jacobs. She brought me into the Nursing and Allied Health Resources Section project on Mapping the Nursing and Allied Health literature with Peg Allen [48] where I mapped Emergency Nursing [49] and Public Health Nursing literature [50].

MLA Special Interest Groups (SIGs) were a place to meet subject matter experts. I joined the SIG on Molecular Biology & Genomics where librarians such as myself, with high school biology and chemistry, connected with information practitioners with graduate degrees in Genetics, Biotechnology, Immunology or other life sciences. Renata McCarthy (now Geer) brought us into the National Center for Biotechnology Information's Educational Collaborative to develop and teach CE workshops. We shared about stories about providing bioinformatics services in *Journal of the Medical Library Association* (JMLA) in 2006 [51].

### Learning through Sharing Knowledge

Teaching CE courses is a great way of learning. I contributed to MLA and the National Library of Medicine's CE courses for Partners in Information Access in Public Health. In 2004, I received a Sewell Foundation stipend to attend the American Public Health Association's annual meeting; this fund supports networking while librarians gain subject matter expertise. I wrote that librarians are powerful contributors to public health in 2007 [52]. Joey Nicholson and I wrote, as a result of our engagement in public health, about pursuing our masters in public health for *MLA News* in 2008 [53].

As I learned more, connections across MLA communities became more apparent: expert searching and grey literature, open access, connecting practitioners to point of care resources, and outreach to unaffiliated practitioners. As Director of the William Rand Kenan, Jr. Library of Veterinary Medicine, I joined the global community of veterinary librarians. The focus on One Health, the interdependence of humans, animals, and the environment, brought my library, public health, and veterinary knowledge base together. In 2009, Carol Vreeland, DVM, MLS, I developed an MLA CE on the Animal-Human Health Knowledge Connection for the Association of North Carolina Health & Science Librarians. The MLA/Medical Informatics Section Career Development Award enabled me to study the intersection between the medical informatics and veterinary communities [54].

### Interprofessional Practice Across Settings

Thinking about where students will practice after graduation and what libraries will support them has always motivated me to partner with other types of libraries. With pharmacists supporting veterinary medical centers who need to learn about animal health and efficiently find veterinary literature, I co-authored an analysis of the veterinary pharmacy literature [55] intended to help pharmacy schools partner with veterinary libraries. With funding from MLA's Ursula Poland Award, I shared findings at the 2018 International Congress for Animal Health Information Specialists.

Changes in libraries have been captured in the names of MLA communities. Public Health/Health Administration dropped Libraries from the name to welcome information professionals regardless of where they worked. The change from Veterinary Medical Libraries to Animal and Veterinary Information Specialists parallels the veterinary medicine accreditation standard language to move from having a physical library to access to human, digital, and physical resources and the information literacy education provided by information professionals.

As a project funded by MLA's then Kronick Traveling Fellowship, I visited five public health libraries in 2005 and all of those had their space repurposed, physical collections were often consolidated into larger health sciences libraries or replaced with digital collections [56]. One of the biggest concerns I have is materials that are only available as leased digital content. The move from owning to leasing core texts is very much a concern in 2024 with discussion posts about the challenges of earlier editions disappearing from packages without notice. It is hard for libraries of public universities serving residents or unaffiliated providers working in the state to ensure access to health knowledge resources.

### Staying Connected Throughout Your Career

As MLA President (2022), I followed all 40-plus MLA caucuses and MEDLIB-L and observed the interconnecting themes. Communities blend contributing and lurking, with a small nucleus actively sharing knowledge or setting up learning activities. They are a benefit of MLA membership and knowing the participants makes it easier and safer to share information on thorny issues. You never truly leave these communities, the knowledge and network keep building on each other like a coral reef expanding on a solid scaffolding in a healthy environment. MLA spawns new knowledge branches as we need them. Today's MLA is greatly extended from medical into the much broader realm of the social determinants of health and often practicing outside of a traditionally defined library.

**Shannon D. Jones, AHIP, FMLA (she/her), 2022–2023 MLA President**

## DIVERSITY, EQUITY, INCLUSION, AND BELONGING IN MLA

Reflecting on the past 25 years of MLA's history, my journey as a Black librarian and champion for diversity, equity, inclusion, and belonging (DEIB) comes into focus. The lens through which I view this period reveals transformative progress toward actualizing its guiding principle: “Diversity, equity, and inclusion are the threads that strengthen the fabric of the Medical Library Association” [57].

In 2002 MLA became my professional home. I was eager to share my time, talent, and unique voice for the greater good of health sciences librarianship. I am proud to witness MLA's efforts to build its DEIB capacity and transform itself to a point where new librarians and Black librarians belong.

### Real Action in Diversity, Equity, Inclusion and Belonging

When I joined MLA, I noted a need and opportunity to foster racial diversity. The pervasiveness of whiteness was palpable at the annual meeting, in the leadership, and on committees. Even in the early 2000s, MLA said it embraced DEIB; however, real action only happened with the appointment of the Diversity and Inclusion Task Force in 2017. An impactful action by the Task Force was to conduct a member survey in 2019 to gather demographic information and member perception of MLA's DEI efforts [58]. Since 2017, meaningful actions have included codifying DEI as a core value, appointing a standing committee, and improving diversity, inclusion, and accessibility in annual meetings [57].

Diversity, equity, and inclusion are critical in promoting a sense of belonging. As members experience a sense of belonging, they see themselves represented and treated fairly; experience acceptance; and gain meaningful connections throughout MLA. In essence, belongingness shapes a member's experience within the association. With this sense of belonging, members will likely maintain their memberships and service to MLA. I posit incorporating belonging into MLA's DEI efforts is necessary to build a more just and equitable experience for everyone.

### African American Medical Librarians Alliance (AAMLA) Caucus

MLA's AAMLA Caucus is my primary professional family. I came into the Association fully aware that Black librarians were underrepresented in the profession and MLA. I wanted to do something about it. AAMLA is where I found my voice, my tribe, and my professional success, thanks, in part, to librarians who came before me. Librarians such as Sandra Franklin, Rosalind Lett, Sandra Martin, Beverly Murphy, Elaine Wells, and other AAMLA members have been outstanding mentors, sponsors, and champions to me in MLA. They encouraged me to run for leadership positions and supported me when I did. AAMLA is where I met lifelong friends and colleagues who share my passion for promoting DEIB and creating environments where Black librarians thrive.

### Belongingness for New Librarians and Members

One of my most meaningful contributions to MLA was in 2005, when I created the New Members Special Interest Group (now Caucus). At the heart of forming the group was cultivating a sense of belonging for new librarians and members. With the encouragement and support of members of AAMLA and the Mid-Atlantic Chapter of MLA, I sought to establish a space for new librarians and members to call their own, where they could feel welcomed, heard, included, and supported. The goal was to provide a forum for members with less than three years of experience to discuss information related to their experience as new librarians and members and to foster a sense of belonging and community. Much of my early leadership experience came from serving as the inaugural convener of this group, which remains a vibrant and vital community to this day.

### Say Their Names

Celebrating MLA's 125th anniversary supports my aim to document the contributions of Black librarians whose contributions have not been acknowledged or celebrated as part of our history. It is imperative that MLA's history includes names and stories of early Black pioneers. Pioneers such as Josephine G. Morton, 62 years before I attended my first MLA meeting, became the first Black librarian to attend an MLA conference in 1940 [59]. Thirty-nine years later, in 1979, Arlee May became the first Black librarian elected to the MLA Board of Directors [60]. That same year, Dr. Gwendolyn S. Cruzat delivered the prestigious Janet Doe Lecture [61], becoming the first Black librarian to do so. I was unaware of these Black trailblazers when I joined MLA. I now recognize my successes in MLA builds upon their pioneering work. I am excited to have witnessed Beverly Murphy become the first Black librarian elected MLA President in 2016. This was a significant milestone in the Association's history [62].

MLA has come a long way with its DEIB efforts, but much work remains. I am confident that we will continue prioritizing belonging to ensure all members feel welcomed, valued, seen, heard, affirmed, and included. We must center the voices and experiences of those historically excluded within MLA. More importantly, in today's climate, we must continue our advocacy for DEIB, not just within MLA but also in our workplaces and communities. I am proud to call MLA my professional home. I look forward to continued collaborations with MLA colleagues to advance DEIB and to build a more just and equitable MLA for all.

## CONCLUSION

As we celebrate the 125th anniversary of MLA, we are grateful for the progress made over the last 25 years and optimistic about our future. We hope this reflection inspires MLA members to contemplate their experiences and contributions to the Association and how those experiences shape our collective future. This writing is also a call to action for MLA members to engage with the issues surrounding the role of health sciences librarians in society and to continue advocating at the national, regional, and local levels. Overall, this compilation is a testament to the resilience and adaptability of the Association and its members as we strive towards a brighter future.
